# Changes in microRNA expression associated with metastasis and survival in patients with uveal melanoma

**DOI:** 10.18632/oncotarget.27559

**Published:** 2020-04-21

**Authors:** Ayushi Vashishtha, Tae Jin Lee, Ashok Sharma, John J. Wallbillich

**Affiliations:** ^1^Center for Biotechnology and Genomic Medicine, Medical College of Georgia, Augusta University, Augusta, GA, USA; ^2^Department of Obstetrics and Gynecology, Medical College of Georgia, Augusta University, Augusta, GA, USA; ^3^Current address: Department of Oncology, Wayne State University College of Medicine, Detroit, MI, USA

**Keywords:** uveal melanoma, metastasis, survival, microRNA, biomarker

## Abstract

Uveal melanoma (UM) is a major intraocular cancer that is molecularly distinct from cutaneous melanoma. Approximately half of patients with UM eventually develop metastasis. The prognosis of metastatic UM is poor, with a median overall survival (OS) of less than a year. In this study, we sought to identify microRNAs (miRNAs) associated with metastasis and OS in UM. We analyzed the miRNA expression and clinical outcomes data from The Cancer Genome Atlas (TCGA) dataset for UM. Differential expression analyses were conducted for each miRNA with respect ever-development of metastasis. Multiple survival analyses were done, using the Cox proportional hazards model, to evaluate interactions between miRNA expression, metastasis, and OS. A total of 22 miRNAs (3 upregulated and 19 downregulated) were differentially expressed between patients with vs. without metastatic UM. These 22 miRNAs could be grouped into four clusters based on similarities in expression patterns. Of the 22 miRNAs differentially expressed with respect to metastasis, 21 were significantly associated with OS. The expression of multiple miRNAs was significantly associated with metastasis and overall survival in patients with UM. Further investigation of these miRNAs as biomarkers and/or therapeutic targets is warranted in the push to improve outcomes for patients with metastatic UM.

## INTRODUCTION

Uveal melanoma (UM) is the most common primary intraocular cancer occurring in adults [1, 2]. The mortality of UM patients is approximately 40–50%; the leading contributor to mortality is development of metastasis, which occurs in up to 50% of UM patients [3, 4]. For patients with metastatic UM, the 1-year survival rate is 20%, the 5-year survival rate is less than 5%, and the median overall survival is only 6–12 months [1, 5–8]. There is no effective therapeutic intervention to treat metastatic UM [4].

While UM may bear histologic resemblance to its more-common cutaneous counterpart, it is considered molecularly distinct. In contrast to cutaneous melanoma, UM has a lower mutational burden [9] and lacks characteristic BRAF and NRAS mutations [9, 10]. Rather, most UM tumors contain GNAQ or GNA11 mutations [11], MAPK pathway activations [12], and cytogenetic abnormalities (monosomy 3 and trisomy 8q) [13].

Given the biologic uniqueness of UM, its propensity to metastasize, and the poor survival outcomes and lack of adequate treatment for metastatic UM, there is a great need to uncover the molecular mechanisms of metastasis in UM and to discover predictive and prognostic biomarkers so as to better optimize understanding and management of this challenging oncologic condition.

Several genetic mutations and gene expression alterations have been associated with the molecular mechanisms responsible for the progression of UM [14]. It has also been shown that epigenetic modifications including microRNAs (miRNAs) are associated with the pathology and progression of UM [14]. miRNAs are small, non-coding RNAs approximately 22 nucleotides in length. Each microRNA can play a crucial role in regulating the expression of multiple genes. miRNAs typically regulate gene expression by altering mRNA stability or repressing translation of mRNA to protein. Several miRNAs play an important role (tumor suppressors or tumor promoters) in cancer development and progression including metastasis [15–20]. However, data are limited regarding the role of miRNAs in metastatic UM [21–23]. Therefore, the purpose of this study was to identify miRNAs associated with UM metastasis and overall patient survival.

## RESULTS

### Demographics, key clinical data, and differences in OS for patients with metastatic vs. non-metastatic UM

**Figure 1:**
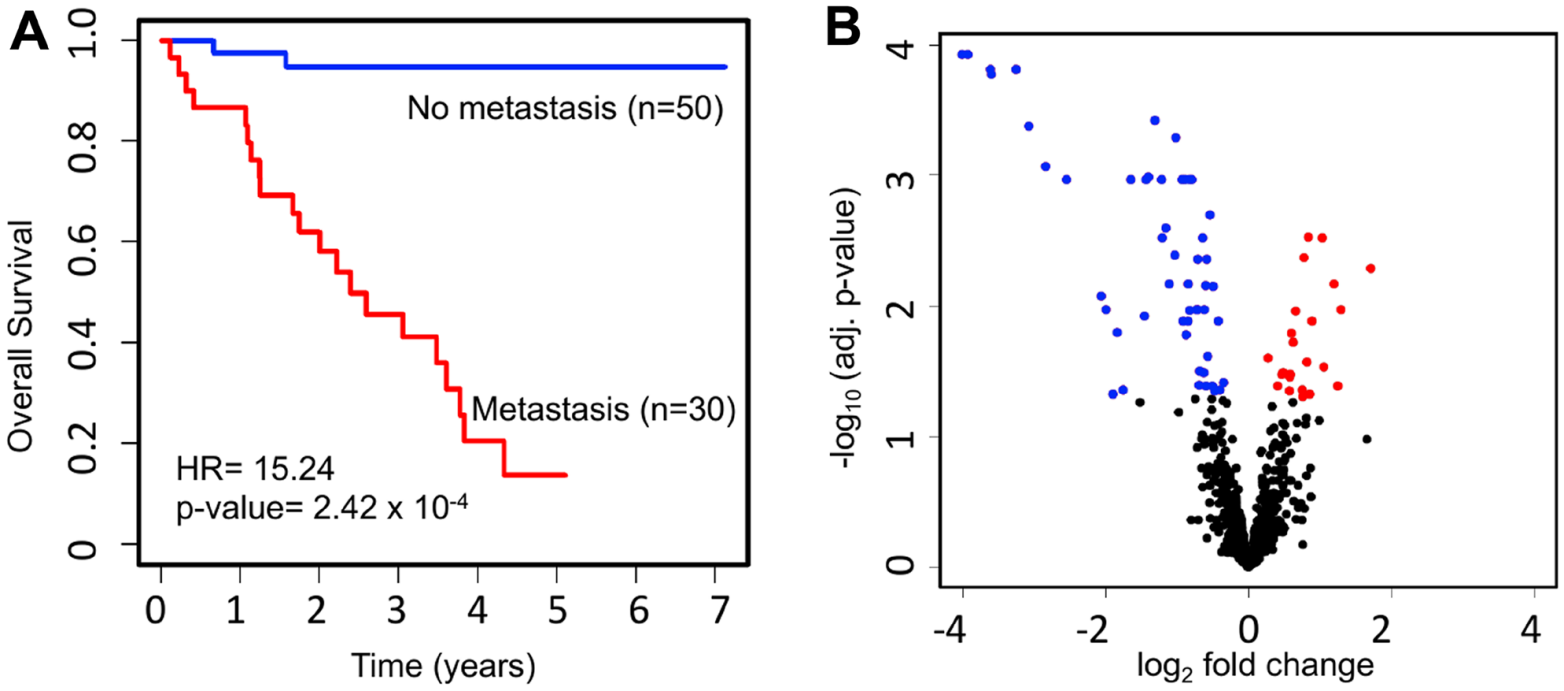
Survival and miRNA expression differences between patients who ever vs. never developed metastatic UM. (**A**) Kaplan-Meier survival curves showing a major difference in OS (HR = 15.24; *p*-value = 2.42 × 10^−4^) for UM patients with (*n* = 30) vs. without (*n* = 50) metastasis. (**B**) Volcano plot depicting differentially expressed miRNAs. The expression level of 1,598 miRNAs was compared between UM patients with metastasis (*n* = 30) and without metastasis (*n* = 50). In total, we discovered 76 significantly dysregulated miRNAs in metastatic patients, including 24 upregulated (red) and 52 downregulated (blue).

Profiling data from 1,598 miRNAs was available for primary-site tumor samples from 80 patients with UM in TCGA. Of those patients, 50 (62.5%) had never developed metastasis, while 30 (37.5%) had ever developed metastasis. Key demographic and clinical data for UM patients with metastatic and non-metastatic UM were identified and compared across the two groups. Compared to those with non-metastatic UM, patients with metastatic UM more often had epithelioid histology, less often had spindle cell histology, and had a higher proportion of advanced (stage III–IV) disease at initial diagnosis (*p*-values all < 0.05). There was no significant difference between non-metastatic and metastatic UM with respect to age, gender, or tumor thickness (Table 1). Survival analysis for metastatic vs. non-metastatic UM showed a major difference (hazard ratio [HR] = 15.24; *p*-value = 2.42 × 10^−4^) with respect to overall survival (OS) between those groups (Figure 1A).

**Table 1:** Demographics and key clinical data for patients with non-metastatic vs. metastatic UM

		Non-Metastatic	Metastatic	
		(*n* = 50)	(*n* = 30)	*p*-value
Age (mean ± SD)		60.65 ± 14.9	64.7 ± 12.12	0.207
Gender	Female	22	13	1.000
	Male	28	17	
Tumor Thickness (mean ± SD)		10.2 ± 2.75	10.78 ± 2.92	0.207
Histologic Type	Epithelioid	4	9	< 0.001
	Mixed epithelioid/spindle	20	17	
	Spindle cell	26	4	
Survival	Survived	48 out 50	9 out of 30	< 0.001
	Deceased	2 out 50	21 out of 30	
Clinical Stage	Stage IIA	2	2	0.023
	Stage IIB	25	7	
	Stage IIIA	14	13	
	Stage IIIB	7	3	
	Stage IIIC	2	1	
	Stage IV	0	4	

### miRNAs differentially expressed in UM with respect to metastasis

**Figure 2:**
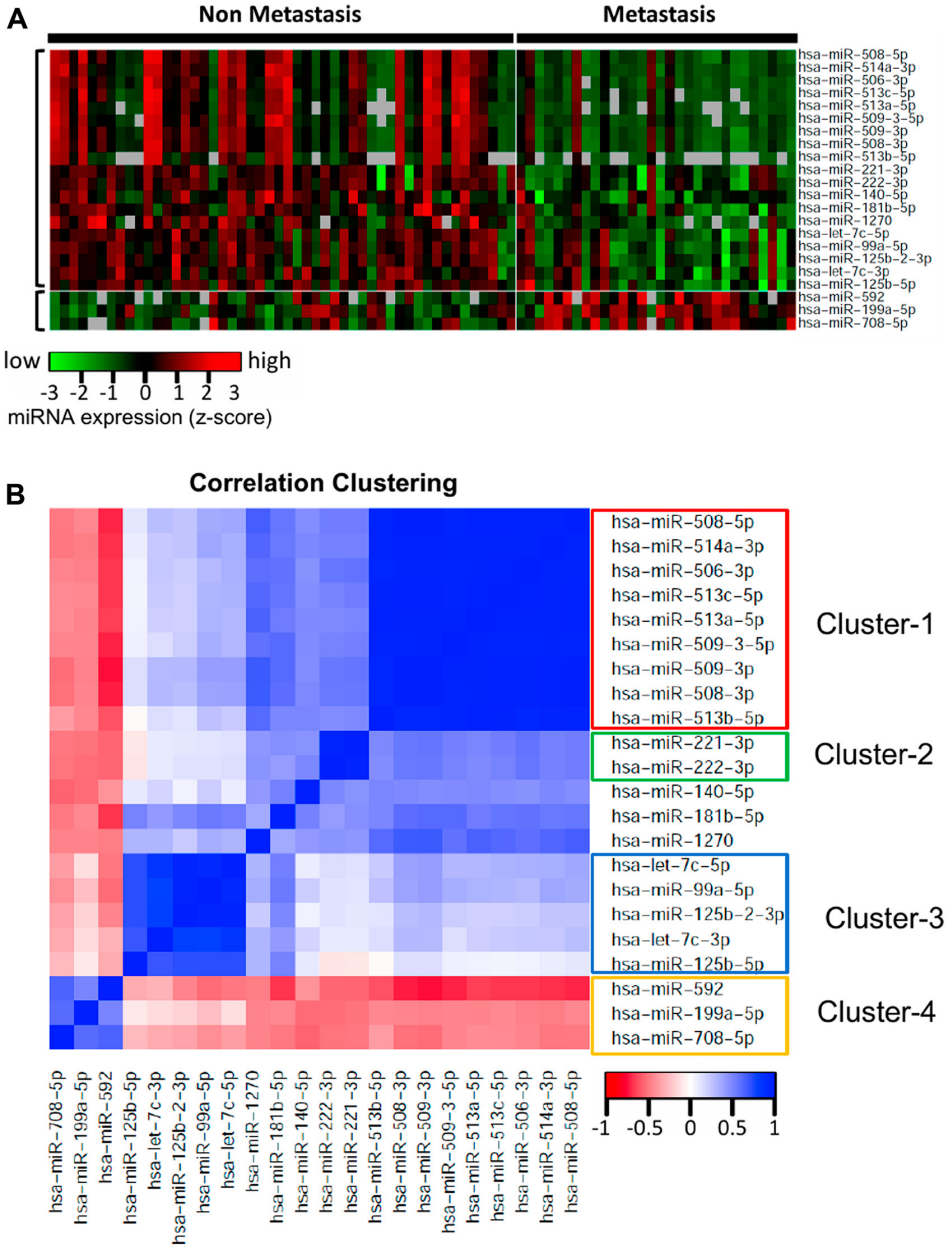
Heatmap and correlation clustering of the miRNAs significantly differentially expressed with respect to ever-development of metastatic UM. (**A**) Heatmap representing the expression of the 22 most highly dysregulated miRNAs in UM metastasis. Out of these 22 miRNAs, 3 are upregulated and 19 are downregulated in patients with metastasis. Each column represents one patient and rows represent miRNAs. (**B**) Pairwise correlations for the 22 miRNAs significantly differentially expressed in UM patients with metastasis. The cluster analysis of the correlation matrix revealed 4 major clusters of highly correlated miRNAs. Cluster-1 included 9 downregulated miRNAs, Cluster-2 included 2 downregulated miRNAs, Cluster-3 included 5 downregulated miRNAs, and Cluster-4 included the 3 upregulated miRNAs.

**Figure 3:**
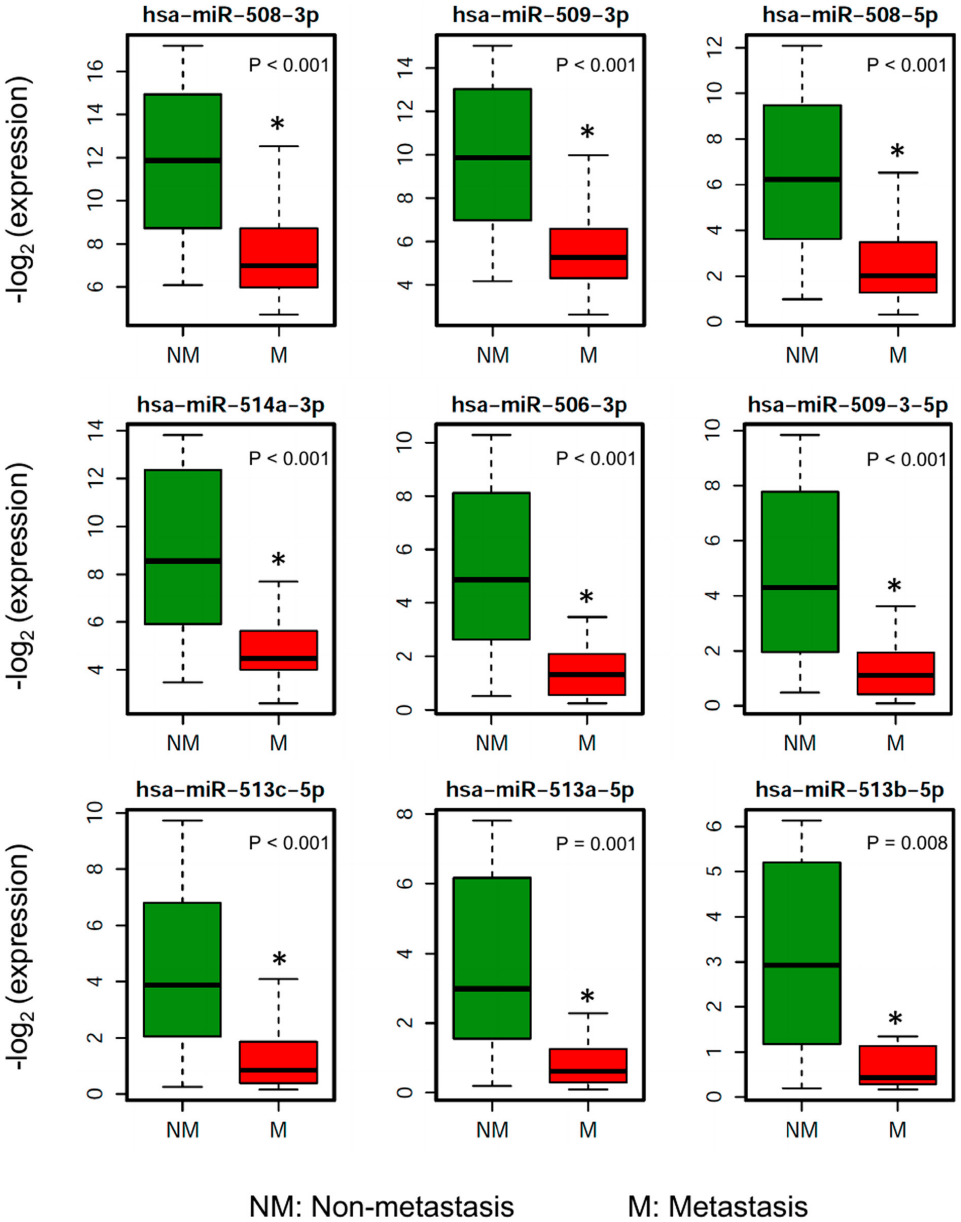
Boxplots showing the distribution of the miRNA expression of the 9 most downregulated miRNAs in patients who ever developed metastasis compared to patients without metastasis.

To investigate the miRNAs associated with UM metastasis, we analyzed the expression of the 1,598 miRNAs available in TCGA UM dataset. Differential expression analysis was done for each of those 1,598 miRNAs between UM patients with (*n* = 30) and without (*n* = 50) metastasis. The volcano plot depicting the results of this differential expression analysis is shown in Figure 1B. In total, we discovered 76 miRNAs (24 upregulated and 52 downregulated) significantly (adj. *p*-value < 0.05) dysregulated in patients with metastasis as compared to patients without metastasis (Supplementary Table 1). The 22 most significantly (> 2-fold change and adj. *p*-value < 0.01) dysregulated miRNAs in patients with metastatic UM are listed in Table 2. A heatmap depicting the expression levels of these 22 miRNAs in individual UM patients is shown in Figure 2A. Out of the 22 miRNAs, 3 are upregulated and 19 are downregulated in patients with metastasis. The upregulated miRNAs in patients with metastasis are miR-199a-5p (3.29-fold), miR-708-5p (2.29-fold), and miR-592 (2.05-fold). Similarly, the most downregulated miRNAs in patients with metastasis are miR-508-3p (0.06-fold), miR-509-3p (0.07-fold), miR-508-5p (0.08-fold), miR-514a-3p (0.08-fold), miR-506-3p (0.11-fold), miR-509-3-5p (0.12-fold), miR-513c-5p (0.14-fold), miR-513a-5p (0.17-fold), and miR-513b-5p (0.24-fold) as shown in Figure 3.

**Table 2:** The 22 most significantly dysregulated miRNAs in patients with metastatic UM

miRNA accession ID	miRNA target name	Fold- change	Adj. *p*-value	HR	Adj. *p*-value	Concordance
Down-regulated miRNAs						
MIMAT0002880	hsa-miR-508-3p	0.062	1.19 × 10^−4^	0.08	4.77 × 10^−7^	0.780
MIMAT0002881	hsa-miR-509-3p	0.065	1.19 × 10^−4^	0.10	3.25 × 10^−6^	0.766
MIMAT0004778	hsa-miR-508-5p	0.081	1.56 × 10^−4^	0.10	2.27 × 10^−6^	0.770
MIMAT0002883	hsa-miR-514a-3p	0.082	1.69 × 10^−4^	0.09	6.38 × 10^−7^	0.778
MIMAT0002878	hsa-miR-506-3p	0.105	1.56 × 10^−4^	0.10	4.88 × 10^−6^	0.760
MIMAT0004975	hsa-miR-509-3-5p	0.118	4.20 × 10^−4^	0.11	1.57 × 10^−5^	0.747
MIMAT0005789	hsa-miR-513c-5p	0.139	8.61 × 10^−4^	0.11	8.88 × 10^−6^	0.752
MIMAT0002877	hsa-miR-513a-5p	0.171	1.09 × 10^-3^	0.09	1.10 × 10^−5^	0.727
MIMAT0005788	hsa-miR-513b-5p	0.239	8.55 × 10^-3^	0.07	8.42 × 10^−5^	0.688
MIMAT0000278	hsa-miR-221-3p	0.319	1.09 × 10^-3^	0.39	0.039	0.607
MIMAT0000279	hsa-miR-222-3p	0.370	1.09 × 10^-3^	0.28	5.09 × 10^–3^	0.645
MIMAT0000097	hsa-miR-99a-5p	0.378	1.03 × 10^-3^	0.24	1.22 × 10^–3^	0.713
MIMAT0000064	hsa-let-7c-5p	0.403	3.83 × 10^−4^	0.27	3.19 × 10^–3^	0.694
MIMAT0005924	hsa-miR-1270	0.430	1.09 × 10^-3^	0.17	1.82 × 10^−4^	0.704
MIMAT0000423	hsa-miR-125b-5p	0.433	3.03 × 10^-3^	0.24	1.31 × 10^–3^	0.711
MIMAT0004603	hsa-miR-125b-2-3p	0.447	2.56 × 10^-3^	0.46	0.075	0.641
MIMAT0026472	hsa-let-7c-3p	0.464	6.82 × 10^-3^	0.28	3.84 × 10^–3^	0.693
MIMAT0000431	hsa-miR-140-5p	0.488	4.13 × 10^-3^	0.15	7.81 × 10^−5^	0.685
MIMAT0000257	hsa-miR-181b-5p	0.494	5.17 × 10^−4^	0.14	3.46 × 10^−5^	0.718
Up-regulated miRNAs						
MIMAT0003260	hsa-miR-592	2.047	3.02 × 10^-3^	4.71	4.49 × 10^−4^	0.723
MIMAT0004926	hsa-miR-708-5p	2.292	6.82 × 10^-3^	4.55	6.28 × 10^−4^	0.682
MIMAT0000231	hsa-miR-199a-5p	3.286	5.15 × 10^-3^	5.50	1.95 × 10^−4^	0.669

### Cluster analysis of miRNAs differentially expressed in UM metastasis

A pairwise correlation was computed for the 22 miRNAs significantly differentially expressed in patients with metastasis. The cluster analysis of the correlation matrix revealed 4 clusters of highly correlated miRNAs (Figure 2B). This analysis provides us the groups of miRNAs which are co-regulated in metastatic UM. Cluster-1 included 9 downregulated miRNAs including miR-508-3p, miR-509-3p, miR-508-5p, miR-514a-3p, miR-506-3p, miR-509-3-5p, miR-513c-5p, miR-513a-5p, and miR-513b-5p. These 9 miRNAs have highly correlated expression levels (average correlation coefficient = 0.95), indicating a probable common regulatory mechanism. Cluster-2 included 2 downregulated miRNAs: miR-221-3p and miR-222-3p. The correlation coefficient between the expression of these miRNAs was 0.92. The Cluster-3 included 5 miRNAs (miR-125b-5p, miR-125b-2-3p, let-7c-3p, miR-140-5p, and miR-181b-5p), which are also downregulated in the patients with metastasis, indicating that these miRNAs have a protective role in UM. Cluster-4 included miR-592, miR-708-5p, and miR-199a-5p, and these 3 miRNAs are upregulated in UM, therefore the miRNAs of this cluster have a negative correlation with the miRNAs of the other three clusters.

### miRNAs associated with survival status in UM

**Figure 4:**
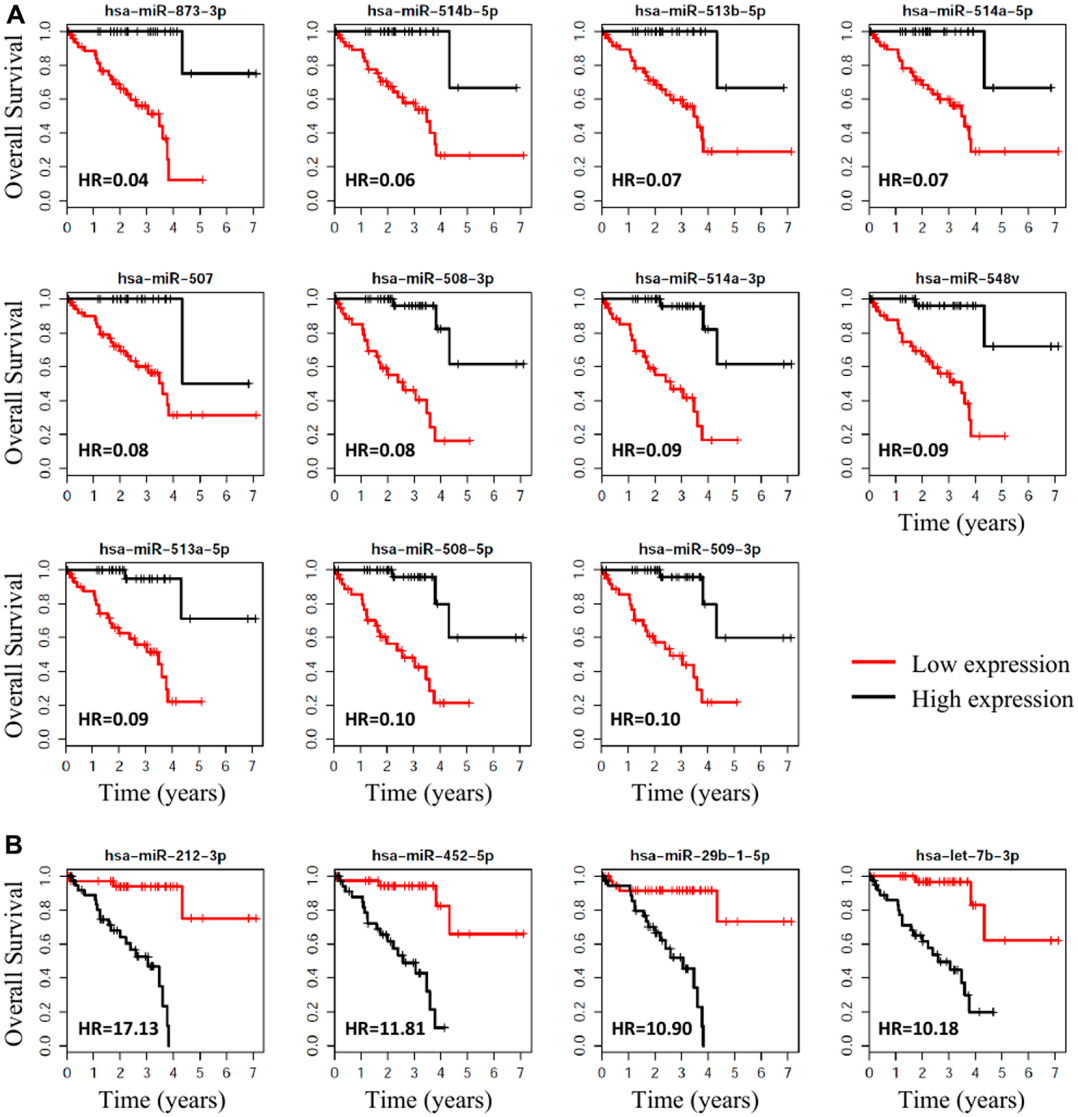
Survival plots, with respect to high vs. low expression, for the miRNAs associated with metastasis that were the most significantly associated with OS for UM. (**A**) The 11 miRNAs that were most (HR < 0.10) protective for OS when highly expressed. (**B**) The 4 miRNAs that were associated with the highest (Hazard Ratio > 10) risk of death.

Cox proportional hazard analysis found that 64 miRNAs were significantly (adj. *p*-value < 0.001 and HR > 4 or HR < 0.2) correlated with patient survival (Supplementary Table 2). The 15 miRNAs most significantly associated with overall survival (OS) of patients (HR > 10 or HR < 0.10) are listed in Table 3; the Kaplan-Meier OS curves of these miRNAs are shown in Figure 4. Of the 22 miRNAs significantly differentially expressed in patients with metastasis, 21 were significantly (*p* < 0.05) associated with OS.

**Table 3:** The 15 miRNAs most significantly associated with overall survival for patients with UM

miRNA accession ID	miRNA target name	HR	Adj. *p*-value	Concordance	Fold change	Adj. *p*-value
miRNAs with Hazard Ratio < 0.10						
MIMAT0022717	hsa-miR-873-3p	0.041	9.73 × 10^−7^	0.721	0.66	0.107
MIMAT0015087	hsa-miR-514b-5p	0.062	2.71 × 10^−5^	0.702	0.25	0.011
MIMAT0005788	hsa-miR-513b-5p	0.070	8.42 × 10^−5^	0.688	0.24	0.009
MIMAT0022702	hsa-miR-514a-5p	0.071	9.56 × 10^−5^	0.686	0.27	0.048
MIMAT0002879	hsa-miR-507	0.082	2.70 × 10^−4^	0.676	0.30	0.045
MIMAT0002880	hsa-miR-508-3p	0.083	4.77 × 10^−7^	0.780	0.06	0.0001
MIMAT0002883	hsa-miR-514a-3p	0.085	6.38 × 10^−7^	0.778	0.08	0.0002
MIMAT0015020	hsa-miR-548v	0.087	1.19 × 10^−5^	0.715	0.53	0.013
MIMAT0002877	hsa-miR-513a-5p	0.087	1.10 × 10^−5^	0.727	0.17	0.001
MIMAT0004778	hsa-miR-508-5p	0.096	2.27 × 10^−6^	0.770	0.08	0.0002
MIMAT0002881	hsa-miR-509-3p	0.099	3.25 × 10^−6^	0.766	0.07	0.0001
miRNAs with Hazard Ratio > 10						
MIMAT0000269	hsa-miR-212-3p	17.126	3.64 × 10^−7^	0.715	1.68	0.045
MIMAT0001635	hsa-miR-452-5p	11.806	1.32 × 10^−6^	0.729	1.60	0.079
MIMAT0004514	hsa-miR-29b-1-5p	10.898	3.80 × 10^−6^	0.681	1.49	0.045
MIMAT0004482	hsa-let-7b-3p	10.184	3.42 × 10^−6^	0.753	1.79	0.003

### Target genes and pathways regulated by miRNAs associated with UM metastasis

**Figure 5:**
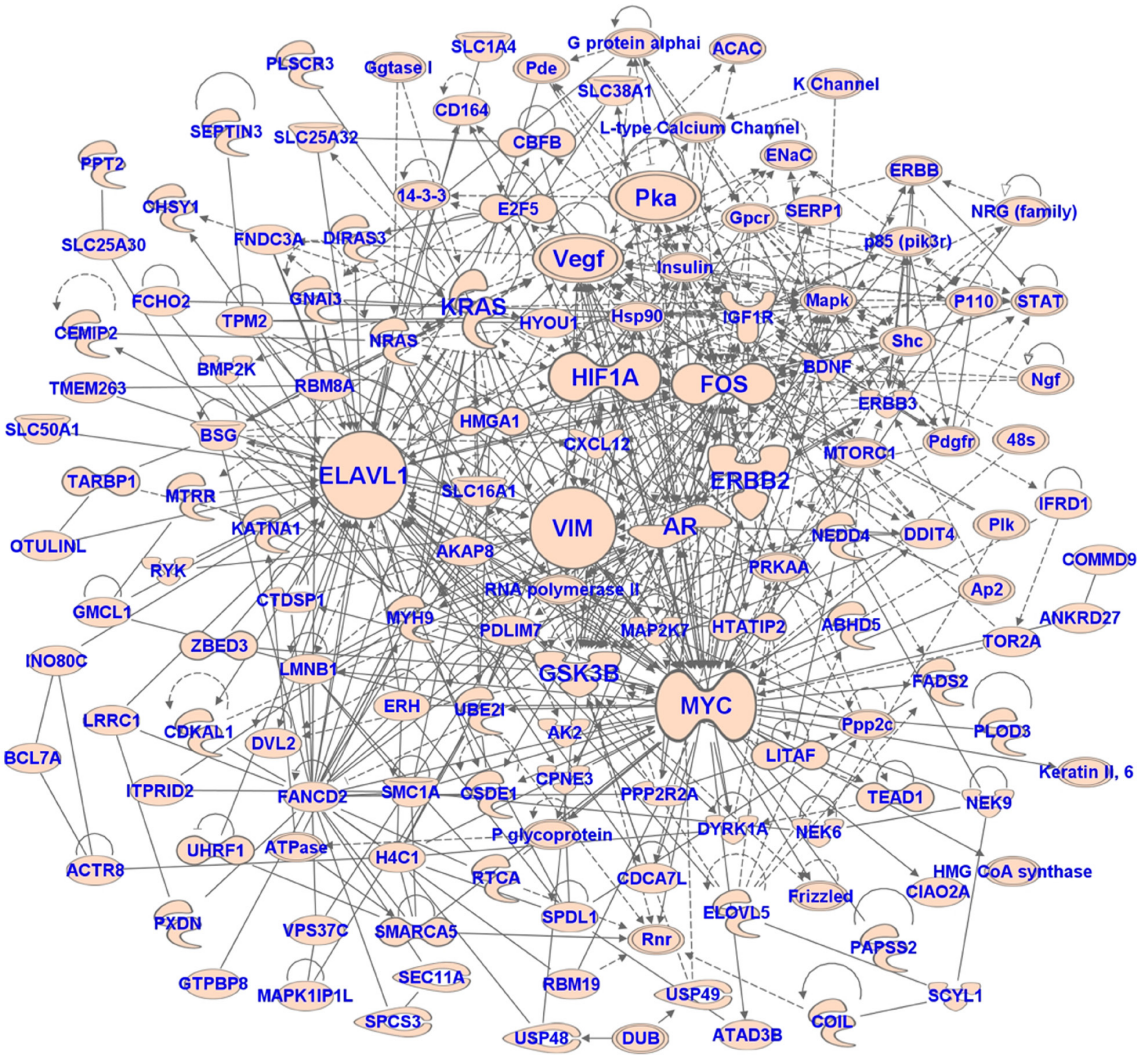
The network of target genes of miRNAs associated with metastasis in UM. The network was generated using IPA software.

A comprehensive search of experimentally validated target genes regulated by miRNAs found to be associated with UM metastasis was performed using Ingenuity Pathway Analysis software (QIAGEN, Redwood City, CA, USA). Further bioinformatics analyses were performed to discover pathways and biological processes associated with these target genes. The top canonical pathways associated with these genes include p53 signaling, regulation of epithelial-mesenchymal transition pathway, cell cycle G1/S checkpoint regulation, ILK signaling, and PTEN signaling (Table 4). The top biological functions include apoptosis, necrosis, growth of tumor, cell proliferation, invasion, movement, migration, and cell cycle progression (Table 4). We also performed network analysis to discover the interactions between the target genes. The top-scoring network is shown in Figure 5. The network analysis revealed that the hub genes of the network are *MYC, VIM, AR, ERBB2, HIF1A, FOS, KRAS, VEGF, PKA, ELAVL1,* and *GSK3B*. These hub genes interact with many (> 15) nodes on the interaction network and are likely important for gene expression dynamics (mechanism). The top upstream regulators of the target genes are *TP53, EGF, TGFB1, PTEN, MYC, SP1, ERBB2, FGF2, HGF, TP63, ESR1, E2F1, KRAS*, and *PI3K* complex (Table 4). These upstream regulators are the predicted transcriptional regulators in the pathway.

**Table 4:** Top canonical pathways, biological functions and upstream regulators

*Canonical pathways*	*p*-value
1. Molecular Mechanisms of Cancer	1.58 × 10^-19^
2. Senescence Pathway	2.51 × 10^-18^
3. p53 Signaling	1.00 × 10^-16^
4. Pancreatic Adenocarcinoma Signaling	1.26 × 10^-14^
5. Glioblastoma Multiforme Signaling	1.00 × 10^-12^
6. Aryl Hydrocarbon Receptor Signaling	3.16 × 10^-12^
7. Regulation of the Epithelial-Mesenchymal Transition Pathway	3.16 × 10^-12^
8. Chronic Myeloid Leukemia Signaling	6.30 × 10^-12^
9. Cell Cycle: G1/S Checkpoint Regulation	7.94 × 10^-12^
10. ILK Signaling	1.99 × 10^-11^
11. PTEN Signaling	2.51 × 10^-11^
12. Colorectal Cancer Metastasis Signaling	3.98 × 10^-11^
13. IL-8 Signaling	5.01 × 10^-11^
14. Glucocorticoid Receptor Signaling	1.94 × 10^-10^
15. Cyclins and Cell Cycle Regulation	1.58 × 10^-09^

*Diseases or functions annotation*	*p*-value
1. Apoptosis	1.43 × 10^-37^
2. Necrosis	1.54 × 10^-37^
3. Growth of tumor	1.65 × 10^-37^
4. Cell proliferation of tumor cell lines	1.89 × 10^-37^
5. Invasion of cells	2.06 × 10^-35^
6. Cell movement of tumor cell lines	3.85 × 10^-34^
7. Migration of cells	1.55 × 10^-32^
8. Growth of malignant tumor	1.76 × 10^-32^
9. Cell movement	7.82 × 10^-32^
10. Migration of tumor cell lines	9.59 × 10^-32^
11. Cell cycle progression	1.68 × 10^-31^
12. Invasion of tumor cell lines	1.97 × 10^-31^

*Upstream regulator*	*Molecule type*	*p*-value
* 1. TP53*	transcription regulator	2.21 × 10^-31^
* 2. EGF*	growth factor	4.00 × 10^-30^
* 3. TGFB1*	growth factor	5.81 × 10^-30^
* 4. PTEN*	phosphatase	1.18 × 10^-29^
* 5. MYC*	transcription regulator	2.56 × 10^-28^
* 6. SP1*	transcription regulator	4.87 × 10^-27^
* 7. ERBB2*	kinase	2.54 × 10^-26^
* 8. FGF2*	growth factor	9.69 × 10^-26^
* 9. HGF*	growth factor	1.66 × 10^-24^
* 10. TP63*	transcription regulator	3.09 × 10^-23^
* 11. ESR1*	ligand-dependent nuclear receptor	4.44 × 10^-23^
* 12. E2F1*	transcription regulator	6.48 × 10^-23^
* 13. KRAS*	enzyme	4.26 × 10^-22^
* 14. PI3K (complex)*	complex	5.69 × 10^-22^

## DISCUSSION

The development of metastases plays an important role in UM patient prognosis. Molecular biomarkers associated with UM metastasis may help in accurately identifying high-risk patients and in discovering potential therapeutic targets for metastatic UM treatment. MicroRNAs are small single-stranded endogenous noncoding RNAs, which are involved in the post-transcriptional regulation of expression of their targeted mRNAs. It has been established that aberrant expression of miRNAs leads to progression and metastasis of several cancers. In the past decade, several studies have examined the role of microRNAs in pathogenesis and progression of UM by utilizing plasma [24], serum [25], cell lines [26–29], and clinical tissue specimens [21–23, 30]. Uveal melanoma is a very rare cancer, making it difficult to obtain a large number of samples from a single institution. TCGA is a landmark cancer genomics dataset which has molecularly characterized cancer and matched normal samples and is an especially important resource for rare cancers such as UM. TCGA also provides data on various clinical and demographic parameters associated with UM and analyzes this larger sample set with careful experimental design and proper control groups. The use of this larger sample size increases the statistical power of the analysis. Also, to minimize the false positives, we have used a very stringent cutoff to select differentially expressed miRNAs. This approach enabled us to identify several novel miRNAs potentially related to UM metastasis which may have clinical, biological, or mechanistic relevance to UM and may expand our understanding of UM tumor progression.

In this study, we found 22 miRNAs highly dysregulated (> 2-fold change and *p* < 0.01) in UM patients with (vs. without) ever-development of metastasis. 21 (95%) of those miRNAs associated with metastasis were also significantly associated with poor OS. The 22 miRNAs associated with metastasis could be divided into four distinct clusters based on highly correlated expression patterns within each cluster.

Cluster-1 included the 9 most downregulated miRNAs including miR-508-3p, miR-509-3p, miR-508-5p, miR-514a-3p, miR-506-3p, miR-509-3-5p, miR-513c-5p, miR-513a-5p, and miR-513b-5p. For miR-508-3p, a recent study showed that decreased expression was significantly associated with metastasis, while overexpression suppressed the epithelial-mesenchymal transition process, in patients with triple-negative breast cancer [31]. Another study found that miR-508-3p and miR-509-3p were downregulated in renal cell carcinoma tissues, while overexpression of those miRNAs suppressed renal cell carcinoma proliferation, invasion, and migration *in vitro* [32]. In ovarian cancer patients, increased expression of miR-508-3p, miR-508-5p, miR-509-3p, and miR-508-5p was correlated with improved clinical outcomes [33]. Data from our study suggests that this miRNA cluster functions as a tumor suppressor as it is markedly downregulated in UM patients with metastasis. Further, the highly correlated expression levels (average correlation coefficient = 0.95) of these 9 miRNAs suggests a probable common regulatory mechanism. Therefore, these miRNAs as well as their target genes may have therapeutic potential to inhibit tumor metastasis and progression.

Cluster-2 included 2 miRNAs, miR-221-3p and miR-222-3p, which were both downregulated in UM patients with metastasis. In a recent study, miR-221-3p and miR-222-3p were also downregulated in gastric cancer cells with high metastatic potential [18].

Cluster-3 contained 5 downregulated miRNAs with respect to metastasis in UM: miR-125b-5p, miR-125b-2-3p, let-7c-3p, miR-140-5p, and miR-181b-5p. Similar to our findings, a recent study also found that miR-140-5p was abnormally downregulated in melanoma tissues and cells [23]. Overexpression of miR-125b-5p inhibited cell proliferation, migration, and invasion in esophageal squamous cell carcinoma [17]. Five hub miRNAs including miR-125b-5p, miR-145-5p, let-7c-5p, miR-218-5p, and miR-125b-2-3p were also found to be related to the prognosis of colorectal cancer [15]. Of these miRNAs, miR-125b-5p and miR-125b-2-3p were also significantly associated in our study as part of the third cluster.

Cluser-4 included the three most significantly upregulated miRNAs, including miR-592, miR-708-5p, and miR-199a-5p. Previous studies have also shown that miR-199a regulates melanoma metastasis related genes and may provide new therapeutic targets [19, 21]. In a recent study, higher expression of miRNA 199a was observed in UM with liver metastasis [22]. Using a genome-wide microarray based approach, another study found that expression of miRNA-199a was one of the most significant discriminators of low metastasis and high metastasis risk of UM patients [21].

The additional bioinformatic analyses we performed identified the target genes and pathways regulated by the miRNAs found to be associated with UM. Several key transcription regulators (TP53, MYC, SP1, TP63, E2F1), growth factors (EGF, TGFB1, FGF2, HGF) and other key regulators including PTEN, ERBB2, ESR1, KRAS and PI3K-complex were found as key targets using this analysis. Previous studies have also reported constitutive activation of these oncogenic pathways in primary UM [34, 35]. Biological functions related to metastasis including cell cycle progression, cell proliferation, invasion, movement and migration were significantly enriched in the target genes.

With the recent advancement of molecular technologies, miRNAs have newfound potential to serve as viable therapeutic tools. Molecular approaches such as AMOs (anti-miR oligonucleotides), LNA anti-miRs, antagomirs, miRNA sponges, and S-miRs (small molecule inhibitors to target specific miRNAs) are available to inhibit the miRNAs overexpressed in cancer [36–40]. On the other hand, molecular approaches to restore the decreased expression of miRNAs downregulated in cancer are also available and include miRNA mimics (double-stranded synthetic RNAs that mimic endogenous miRNAs) and miRNA expression vectors. Several studies have used miRNA replacement therapy in experimental models [41–43]. Additionally, in a recent study, aptamer-miRNA conjugates were used as a novel tool for targeted delivery of miRNAs [44]. Several miRNA-based therapies are already in clinical trials, for example, miR-16 mimics are under phase 1 clinical trials for patients with recurrent thoracic cancer [45].

A major limitation of this study was a lack of experimental validation of the findings with either a separate dataset from patient samples or through *in vivo* or *in vitro* experiments. Further, as with other investigations primarily based on data from TCGA, our analyses used retrospectively obtained data and TCGA UM patient population may not be fully generalizable to some UM patient populations with demographic or clinical features under-represented in TCGA.

In conclusion, this study identified, in primary-site tumor samples, altered miRNA expression patterns associated with ever-development of metastasis in patients with uveal melanoma. We found several known tumor suppressor miRNAs to be downregulated in UM patients with metastasis. These results support the increasingly accepted concept that miRNAs play a major role in metastasis. Our finding of 95% overlap between (a) miRNAs associated with UM metastasis and (b) miRNAs associated with poor survival in patients with UM warrants further investigation those overlapping miRNAs. Future evaluation of the 21 overlapping miRNA as prognostic biomarkers and/or therapeutic targets may be a step toward improved outcomes for those with metastatic UM, a patient population that suffers from high mortality and a lack of effective treatment options.

## MATERIALS AND METHODS

### Dataset

The Cancer Genome Atlas (TCGA; RRID: SCR_003193) is one of the foremost data repositories providing molecular characterization of more than 20,000 primary cancers, including unprecedented amounts of miRNA sequence data (~11,000 libraries) across 33 cancer types [20]. We therefore chose TCGA as the dataset for this investigation. We utilized this high-quality data for our study to analyze the differential miRNA expression between patients with and without metastasis. For the sake of this analysis, “metastasis” refers to metastasis at either initial presentation/diagnosis or recurrence (i. e., ever-development of metastasis).

TCGA miRNA expression data was generated using the Illumina HiSeq/GA miRseq and was reported as counts normalized to reads per million mapped reads (RPM). The uveal melanoma miRNA dataset was downloaded from UCSC Xena browser [46].

The dataset includes miRNA expression data from each sampled tumor, as well as corresponding demographic and clinical information such as patient survival and presence of metastases. For statistical analyses, expression values were log2 transformed to achieve a normal distribution. All statistical analyses were performed using the R language and environment for statistical computing (R version 3.5.2; R Foundation for Statistical Computing; https://www.r-project.org; RRID: SCR_001905).

### Differential expression analysis

miRNA expression observations were normalized and differential miRNA expression between metastatic and non-metastatic tumors was analyzed for all evaluable miRNAs in TCGA UM dataset using the LIMMA package (RRID: SCR_010943) [47]. *P*-values were adjusted using the false discovery rate (FDR) method. Also, to minimize the false positives, a cut-off of fold change > 2 and adj. *p*-value < 0.01 was used to select the differentially expressed miRNAs.

### Survival analysis

The survival difference between patients with vs. without metastatic UM in this TCGA dataset was calculated using the Cox proportional hazard model [48]. Independently, we performed survival analyses for each miRNA in the UM TCGA dataset. For each miRNA, subjects were separated into high-expression or low-expression groups relative to the median expression value. Cox proportional hazard models were fitted for each miRNA. The *p*-values for HRs were computed and adjusted using the FDR method.

### Bioinformatics analyses

Ingenuity Pathway Analysis (IPA) software was used to identify the target genes of miRNAs found to be associated with UM metastasis. Bioinformatics analyses of the target genes were performed using the IPA software for identification of enriched canonical pathways and biological functions. The prediction of upstream regulators was also done using the IPA software.

## SUPPLEMENTARY MATERIALS


