# Supplementary material for: Changes in microRNA expression associated with metastasis and survival in patients with uveal melanoma
Source: Oncotarget. 2020 Apr 21;11(16):1435–47. doi: 10.18632/oncotarget.27559 (PMC7185062; doi:10.18632/oncotarget.27559)
Supplement: Supplementary file 1 [file oncotarget-11-1435-s001.pdf]

Changes in microRNA expression associated with metastasis and survival in patients with uveal melanoma

SUPPLEMENTARY MATERIALS

Supplementary Table 1: The 76 miRNAs (52 downregulated and 24 upregulated) significantly (adj. *p*-value < 0.05) dysregulated in patients with ever- vs never-development of metastatic UM

miRNA	Fold change	Adj. *P*-val	HR	Adj. *P*-Val	Concordance
hsa-miR-508-3p	0.0618	0.000119	0.0832	4.77E-07	0.779586
hsa-miR-509-3p	0.0654	0.000119	0.0991	3.25E-06	0.766272
hsa-miR-508-5p	0.0814	0.000156	0.0959	2.27E-06	0.769724
hsa-miR-514a-3p	0.0820	0.000169	0.0853	6.38E-07	0.777613
hsa-miR-506-3p	0.1045	0.000156	0.1026	4.88E-06	0.760355
hsa-miR-509-3-5p	0.1183	0.000420	0.1139	1.57E-05	0.746548
hsa-miR-513c-5p	0.1394	0.000861	0.1081	8.88E-06	0.751972
hsa-miR-513a-5p	0.1706	0.001085	0.0872	1.10E-05	0.727318
hsa-miR-513b-5p	0.2393	0.008545	0.0700	8.42E-05	0.687870
hsa-miR-514b-5p	0.2505	0.010719	0.0616	2.71E-05	0.701677
hsa-miR-514a-5p	0.2678	0.047475	0.0710	9.56E-05	0.686391
hsa-miR-509-5p	0.2785	0.015970	0.1831	0.0040337	0.652860
hsa-miR-507	0.2959	0.044533	0.0815	0.0002689	0.676036
hsa-miR-221-3p	0.3188	0.001085	0.3902	0.0387453	0.606509
hsa-miR-187-3p	0.3637	0.012001	0.3588	0.0181841	0.593688
hsa-miR-222-3p	0.3700	0.001085	0.2752	0.0050915	0.644970
hsa-miR-99a-5p	0.3779	0.001031	0.2354	0.0012149	0.713018
hsa-let-7c-5p	0.4025	0.000383	0.2686	0.0031879	0.693787
hsa-miR-1270	0.4297	0.001085	0.1691	0.000182	0.704142
hsa-miR-125b-5p	0.4328	0.003025	0.2371	0.0013117	0.711045
hsa-miR-125b-2-3p	0.4475	0.002557	0.4639	0.0748833	0.641026
hsa-let-7c-3p	0.4636	0.006818	0.2755	0.0038437	0.693294
hsa-miR-140-5p	0.4881	0.004127	0.1506	7.81E-05	0.685404
hsa-miR-181b-5p	0.4935	0.000517	0.1413	3.46E-05	0.718442
hsa-miR-140-3p	0.5247	0.001085	0.1549	7.66E-05	0.697732
hsa-miR-548v	0.5284	0.013173	0.0872	1.19E-05	0.715483
hsa-miR-222-5p	0.5397	0.001085	0.4127	0.0492873	0.602564
hsa-miR-211-5p	0.5443	0.016864	0.3406	0.0158004	0.654339
hsa-miR-30c-2-3p	0.5552	0.013173	0.1431	4.47E-05	0.724359
hsa-miR-1249-3p	0.5562	0.006818	0.4937	0.1028749	0.586785
hsa-miR-181a-5p	0.5639	0.010790	0.1946	0.0002326	0.714990
hsa-miR-197-3p	0.5641	0.001085	0.3150	0.0079138	0.654832
hsa-miR-221-5p	0.5756	0.001085	0.2650	0.0038028	0.660750

hsa-miR-219a-1-3p	0.6036	0.010719	0.2373	0.001166	0.674556
hsa-miR-29b-2-5p	0.6099	0.004437	0.3885	0.0318223	0.629684
hsa-miR-99a-3p	0.6099	0.010719	0.3407	0.0145477	0.633136
hsa-miR-3125	0.6194	0.040904	0.5286	0.1890491	0.583826
hsa-miR-192-5p	0.6228	0.031757	0.2600	0.0032698	0.623274
hsa-miR-28-5p	0.6397	0.003024	0.2460	0.0015655	0.675542
hsa-miR-1296-5p	0.6480	0.032880	0.1599	8.83E-05	0.696746
hsa-miR-28-3p	0.6511	0.010719	0.2741	0.0036688	0.662229
hsa-miR-2116-3p	0.6601	0.007039	0.1903	0.0002678	0.701677
hsa-miR-29c-5p	0.6610	0.041240	0.5290	0.1344967	0.592702
hsa-miR-942-5p	0.6669	0.004437	0.3156	0.0092858	0.656312
hsa-miR-6509-5p	0.6742	0.024332	0.5130	0.1189007	0.611440
hsa-let-7g-5p	0.6879	0.002035	0.3439	0.014441	0.621302
hsa-miR-574-3p	0.7037	0.041240	0.3390	0.0162342	0.636588
hsa-miR-191-5p	0.7095	0.007149	0.3193	0.0087945	0.645957
hsa-miR-191-3p	0.7194	0.045199	0.2822	0.003567	0.668639
hsa-miR-1910-5p	0.7448	0.013173	0.2266	0.002133	0.664201
hsa-miR-26a-5p	0.7568	0.044533	0.5608	0.1718813	0.610947
hsa-miR-6802-3p	0.7836	0.038942	0.4293	0.0521524	0.622288
hsa-miR-4740-5p	1.2084	0.025263	3.2453	0.0055337	0.650394
hsa-miR-29a-5p	1.3259	0.041240	2.4858	0.0379592	0.602564
hsa-miR-27b-5p	1.3823	0.033895	2.3457	0.0454256	0.590730
hsa-miR-24-2-5p	1.3981	0.032880	3.1377	0.0099514	0.631164
hsa-miR-29b-1-5p	1.4877	0.044972	10.8980	3.80E-06	0.680966
hsa-let-7a-3p	1.4930	0.035239	5.0801	0.0004453	0.707101
hsa-miR-887-5p	1.4988	0.033895	4.3956	0.0008044	0.635108
hsa-miR-212-5p	1.5146	0.016335	7.6168	2.44E-05	0.709566
hsa-miR-4709-3p	1.5421	0.019107	5.3379	0.0002386	0.664694
hsa-miR-766-3p	1.5817	0.011075	1.7536	0.1841243	0.567554
hsa-miR-212-3p	1.6832	0.044509	17.1257	3.64E-07	0.714990
hsa-miR-224-5p	1.6924	0.049509	4.8198	0.0005809	0.645957
hsa-miR-4661-5p	1.7160	0.004279	3.7805	0.0043129	0.602564
hsa-miR-21-3p	1.7621	0.027085	3.0823	0.0125988	0.619329
hsa-let-7b-3p	1.7873	0.002979	10.1842	3.42E-06	0.753452
hsa-miR-887-3p	1.8143	0.047443	6.3928	8.42E-05	0.669625
hsa-miR-145-5p	1.8534	0.013173	3.7098	0.0041597	0.619822
hsa-miR-592	2.0466	0.003024	4.7104	0.0004486	0.723373
hsa-miR-708-3p	2.0767	0.029427	2.6077	0.0254795	0.630178
hsa-miR-708-5p	2.2915	0.006818	4.5538	0.0006277	0.681953
hsa-miR-199b-3p	2.3736	0.041496	6.9885	4.48E-05	0.686884
hsa-miR-199a-3p	2.3782	0.041240	6.9885	4.48E-05	0.686884
hsa-miR-155-5p	2.4541	0.010719	5.4935	0.0002855	0.673570
hsa-miR-199a-5p	3.2856	0.005153	5.4961	0.0001946	0.668639

Supplementary Table 2: The 64 miRNAs significantly (adj. *p*-value < 0.001 and HR > 2 or HR < 0.2) correlated with survival in patients with UM

miRNA	HR	Adj. *P*-Val.	Concordance	Fold change	Adj. *P*-Val.
hsa-miR-873-3p	0.0408	9.732E-07	0.7214004	0.655	0.10666
hsa-miR-514b-5p	0.0616	2.708E-05	0.7016765	0.250	0.01072
hsa-miR-513b-5p	0.0700	8.424E-05	0.6878698	0.239	0.00855
hsa-miR-514a-5p	0.0710	9.565E-05	0.6863905	0.268	0.04747
hsa-miR-507	0.0815	2.689E-04	0.6760355	0.296	0.04453
hsa-miR-508-3p	0.0832	4.770E-07	0.7795858	0.062	0.00012
hsa-miR-514a-3p	0.0853	6.383E-07	0.7776134	0.082	0.00017
hsa-miR-548v	0.0872	1.190E-05	0.7154832	0.528	0.01317
hsa-miR-513a-5p	0.0872	1.102E-05	0.7273176	0.171	0.00108
hsa-miR-508-5p	0.0959	2.266E-06	0.7697239	0.081	0.00016
hsa-miR-509-3p	0.0991	3.249E-06	0.7662722	0.065	0.00012
hsa-miR-935	0.1012	4.803E-06	0.7465483	0.508	0.06541
hsa-miR-506-3p	0.1026	4.875E-06	0.7603550	0.105	0.00016
hsa-miR-1468-5p	0.1047	1.310E-05	0.7140039	0.713	0.21847
hsa-miR-873-5p	0.1053	1.020E-05	0.7317554	0.652	0.11593
hsa-miR-513c-5p	0.1081	8.879E-06	0.7519724	0.139	0.00086
hsa-miR-509-3-5p	0.1139	1.573E-05	0.7465483	0.118	0.00042
hsa-miR-510-3p	0.1383	5.473E-04	0.6952663	0.349	0.05493
hsa-miR-181b-5p	0.1413	3.457E-05	0.7184418	0.494	0.00052
hsa-miR-30c-2-3p	0.1431	4.466E-05	0.7243590	0.555	0.01317
hsa-miR-140-5p	0.1506	7.810E-05	0.6854043	0.488	0.00413
hsa-miR-140-3p	0.1549	7.658E-05	0.6977318	0.525	0.00108
hsa-miR-1296-5p	0.1599	8.825E-05	0.6967456	0.648	0.03288
hsa-miR-181a-2-3p	0.1659	8.497E-05	0.7140039	0.639	0.09753
hsa-miR-1270	0.1691	1.820E-04	0.7041420	0.430	0.00108
hsa-miR-4797-3p	0.1795	2.895E-04	0.6829389	0.746	0.08162
hsa-miR-181c-3p	0.1854	3.070E-04	0.6962525	0.910	0.78529
hsa-miR-2116-3p	0.1903	2.678E-04	0.7016765	0.660	0.00704
hsa-miR-26a-1-3p	0.1907	2.749E-04	0.6829389	0.894	0.56829
hsa-miR-181a-5p	0.1946	2.326E-04	0.7149901	0.564	0.01079
hsa-miR-4758-5p	4.1129	9.661E-04	0.6932939	1.150	0.48591
hsa-miR-1228-5p	4.3078	9.334E-04	0.6208087	1.065	0.66881
hsa-miR-887-5p	4.3956	8.044E-04	0.6351085	1.499	0.03389
hsa-miR-502-5p	4.4155	6.331E-04	0.7214004	1.192	0.41127
hsa-miR-708-5p	4.5538	6.277E-04	0.6819527	2.292	0.00682
hsa-miR-150-5p	4.7026	8.341E-04	0.6173570	1.619	0.35387
hsa-miR-592	4.7104	4.486E-04	0.7233728	2.047	0.00302
hsa-miR-193b-3p	4.7334	5.356E-04	0.6637081	1.411	0.18159
hsa-miR-214-5p	4.7578	4.678E-04	0.6637081	1.091	0.66326
hsa-miR-224-5p	4.8198	5.809E-04	0.6459566	1.692	0.04951
hsa-miR-132-5p	4.9098	7.393E-04	0.6533531	1.420	0.08279
hsa-miR-188-5p	5.0075	4.206E-04	0.6696252	1.346	0.15694
hsa-miR-146b-5p	5.0696	3.423E-04	0.6676529	1.361	0.45453

hsa-let-7a-3p	5.0801	4.453E-04	0.7071006	1.493	0.03524
hsa-miR-452-3p	5.2700	1.491E-04	0.7154832	1.056	0.62800
hsa-miR-937-3p	5.2976	3.171E-04	0.6780079	1.754	0.07247
hsa-miR-195-5p	5.3196	1.655E-04	0.6849112	1.431	0.14561
hsa-miR-4709-3p	5.3379	2.386E-04	0.6646943	1.542	0.01911
hsa-miR-200b-3p	5.4695	1.843E-04	0.6942801	1.392	0.09753
hsa-miR-155-5p	5.4935	2.855E-04	0.6735700	2.454	0.01072
hsa-miR-199a-5p	5.4961	1.946E-04	0.6686391	3.286	0.00515
hsa-miR-103a-2-5p	6.2733	5.394E-05	0.6923077	1.234	0.09125
hsa-miR-887-3p	6.3928	8.417E-05	0.6696252	1.814	0.04744
hsa-miR-199a-3p	6.9885	4.479E-05	0.6868836	2.378	0.04124
hsa-miR-199b-3p	6.9885	4.479E-05	0.6868836	2.374	0.04150
hsa-miR-501-5p	7.0069	4.275E-05	0.6829389	1.102	0.69545
hsa-miR-497-5p	7.1473	3.608E-05	0.7258383	1.348	0.18466
hsa-miR-212-5p	7.6168	2.436E-05	0.7095661	1.515	0.01633
hsa-miR-155-3p	8.1201	4.544E-06	0.7120316	1.097	0.75543
hsa-miR-148b-3p	8.1465	3.530E-05	0.6799803	1.186	0.23882
hsa-let-7b-3p	10.1842	3.421E-06	0.7534517	1.787	0.00298
hsa-miR-29b-1-5p	10.8980	3.800E-06	0.6809665	1.488	0.04497
hsa-miR-452-5p	11.8063	1.324E-06	0.7287968	1.601	0.07938
hsa-miR-212-3p	17.1257	3.642E-07	0.7149901	1.683	0.04451
